# Population Pharmacogenetic-Pharmacokinetic Approach in the Context of Methadone Administration in Galicia, Spain

**DOI:** 10.1192/j.eurpsy.2026.10499

**Published:** 2026-07-31

**Authors:** J. Gomez-Trigo, S. Recarey-Rama, A. Gil Rodríguez, A. Rodríguez-Viyuela, R. Cruz-Guerrero, A. M. Barral-Raña, E. Domínguez-Medina, M. Arrojo-Romero, O. Maroñas-Amigo

**Affiliations:** 1 Servicio de Psiquiatría, Complejo Hospitalario Universitario de Santiago de Compostela; 2 Grupo de Farmacogenómica y Descubrimiento de Medicamentos, Fundación Instituto de Investigación Sanitaria de Santiago de Compostela (IDIS); 3 Grupo de Medicina Genómica, Centro de Investigación en Medicina Molecular y Enfermedades Crónicas (CiMUS), Santiago de Compostela, Spain

## Abstract

**Introduction:**

Methadone is a synthetic opioid with slow-onset activity, acting as an agonist at μ-opioid receptors (MOR) and as an antagonist at N-methyl-D-aspartate (NMDA) receptors. Its biotransformation is primarily mediated by cytochrome P450 enzymes, including CYP3A4, CYP2C19, CYP2D6, and particularly CYP2B6, which exhibits preferential metabolism of the R-enantiomer. Single nucleotide polymorphisms (SNPs) have been shown to influence methadone pharmacokinetics and contribute to interindividual variability. Owing to this metabolic variability, a consistent correlation between dose, plasma concentration, and clinical effect has not been established, thereby necessitating individualized dose titration guided by clinical response.

**Objectives:**

This study aims to elucidate the relationship between plasma methadone concentrations and patient-specific variables, including body weight, sex, and metabolizer phenotypes of the principal genes implicated in methadone biotransformation.

**Methods:**

This descriptive study involved genotyping and phenotyping a cohort of 80 patients enrolled in methadone substitution programs in the Galician community. Two blood samples were collected from each participant: one to determine the metabolizer phenotype for the previously mentioned enzymes, and another to isolate plasma for quantification of methadone concentrations.

**Results:**

Following pharmacogenetic and pharmacokinetic analyses, significant differences were observed between normal and intermediate CYP3A4 metabolizers (p = 0.0327), whereas no significant associations were found for the other enzymes, supporting the hypothesis that the enzyme encoded by this gene is the predominant contributor.

**Conclusions:**

Although no correlation was detected between methadone metabolism and BMI, body weight, or sex, the observed association between CYP3A4 metabolizer status and plasma methadone concentrations highlights the potential for precision medicine approaches in guiding the prescription of methadone for opioid use disorder.

**Disclosure of Interest:**

None Declared

